# KIF6 719Arg Carrier Status Association with Homocysteine and C-Reactive Protein in Amnestic Mild Cognitive Impairment and Alzheimer's Disease Patients

**DOI:** 10.1155/2013/242303

**Published:** 2013-12-24

**Authors:** Michael Malek-Ahmadi, Amar Patel, Marwan N. Sabbagh

**Affiliations:** Banner Sun Health Research Institute, Cleo Roberts Center for Clinical Research, Sun City, AZ 85351, USA

## Abstract

Recent research has demonstrated associations between statin use, KIF6 719Arg carrier status, and cholesterol levels and amnestic mild cognitive impairment (aMCI) and Alzheimer's disease (AD) patients. The association between 719Arg carrier status with homocysteine (tHcy) and c-reactive protein (CRP) levels in aMCI and AD has not been previously investigated. Data from 175 aMCI and AD patients were used for the analysis. 719Arg carriers had significantly lower levels of tHcy than noncarriers (*P* = 0.02). No significant difference in CRP levels between 719Arg carriers and noncarriers was present (*P* = 0.37). Logistic regression yielded no significant effect for 719Arg status on CRP [OR = 1.79 (0.85, 3.83), *P* = 0.13] but did demonstrate a significant effect for tHcy [OR = 0.44 (0.23, 0.83), *P* = 0.01] after adjusting for ApoE *ε*4 carrier status, age, gender, and statin use. This study is the first to explore the relationship between KIF6 719Arg carrier status with tHcy and CRP levels. 719Arg carriers were more likely to have normal tHcy levels after adjusting for ApoE *ε*4 status, age, gender, and statin use. These results suggest that the KIF6 gene might influence cardiovascular pathways associated with AD.

## 1. Introduction

The KIF6 gene is one of several molecular components involved in the intracellular transport of protein complexes, membrane organelles, and messenger ribonucleic acid along microtubules [1]. There are three polymorphisms of the KIF6 gene which include Arg/Arg, Trp/Arg, and Trp/Trp. Individuals carrying the Arg/Arg or Trp/Arg polymorphisms are classified as 719Arg carriers and have been linked to an increased risk for cardiovascular disease [1–6] relative to 719Arg noncarriers (Trp/Trp). Some of these studies also demonstrated a greater lipid lowering response from statin therapy among 719Arg carriers when compared to noncarriers [1–4]. Others have found no association between 719Arg carrier status and cardiovascular outcomes [7, 8]. A recent study by Sabbagh et al. [9] found that positive 719Arg carrier status combined with statin use was associated with lower total cholesterol (TC) and lower low density lipoprotein (LDL) levels in a sample of amnestic mild cognitive impairment (aMCI) and Alzheimer's disease (AD) patients.

In terms of disease risk, cholesterol levels and statin use have garnered significant attention from aMCI/AD researchers [10–13]; however, homocysteine (tHcy) [14, 15] and c-reactive protein (CRP) [16–18] have also been implicated as risk factors for aMCI and AD. Troen and Rosenberg [19] describe several studies that established elevated tHcy as a risk factor for AD; however, several studies which found no association with tHcy and AD are also cited. Previous research has shown that elevated tHcy may lead to intraneuronal accumulation of A*β*42 as a result of neurotoxicity which inhibits cellular DNA repair mechanisms [20]. Irizarry et al. [21] found that circulating tHcy correlated moderately with circulating A*β*42 but concluded that elevated tHcy was not associated with AD or aMCI status.

Elevated CRP has also been established as a risk factor for AD [16–18] and is thought to be associated with inflammatory pathways involved with AD pathogenesis [22]. Bi et al. [23] found that both *in vivo* and *in vitro* CRP cytotoxicity were positively correlated with A*β* formation while Kok et al. [24] demonstrated that allelic variations in the CRP gene are associated with differing levels senile plaque formation. In contrast, O'Bryant et al. [25] found that AD patients had significantly lower CRP levels relative to controls while Roberts et al. [26] found no association with elevated CRP and amnestic MCI [OR = 1.21; 95% CI (0.81, 1.82)]. Haan et al. [27] found that ApoE *ε*4 carriers had significantly lower CRP levels than noncarriers and that higher CRP levels were associated with a decreased risk of incident all-cause CIND/dementia among ApoE *ε*4 carriers [OR = 0.60; 95% CI (0.20, 0.91) *P* = 0.03]. Among cognitively normal older adults, Ravaglia et al. [28] found that ApoE *ε*4 carriers with normal CRP levels had a significantly lower risk of elevated tHcy levels [OR = 0.22; 95% CI (0.08, 0.59)] and were less likely to have elevated CRP levels in the presence of normal tHcy levels [OR = 0.51; 95% CI (0.31, 0.85)] when compared to noncarriers.

Since tHcy and CRP have demonstrated associations with cardiovascular disease [29, 30] and AD [14–18], we sought to investigate whether KIF6 719Arg carrier status is associated with tHcy and CRP in a group of clinically diagnosed AD and aMCI patients. Since KIF6 719Arg carrier status has been previously associated with cardiovascular disease [1–6], investigating its associations with tHcy and CRP in AD is of interest given the proposed cardiovascular pathways for AD pathogenesis [31, 32].

## 2. Method 

### 2.1. Study Sample

Data from 175 patients (74 aMCI, 101 AD) between the ages of 53 and 97 who were seen in a neurology clinic in Sun City, AZ, were used for the analysis. All patients were of Caucasian ethnicity. Clinical diagnoses of aMCI and probable or possible AD were made based on medical history, social history, clinical laboratory results, mental status exam, assessment of daily functioning, MRI, and neuropsychological testing. The AD patients met NINCDS-ADRDA [33] criteria for a clinical diagnosis of probable or possible AD. Petersen criteria were used to classify aMCI patients [34]. This study was exempted from review by the Western IRB as it was a study that utilized existing data which was recorded in a way that prevented the identities of the patients from being known.

### 2.2. CRP, tHcy, KIF6 Genotype, and ApoE Genotype Processing

CRP was processed with an automated immunoturbidimetric assay using Roche Modular and tHcy was processed with an enzymatic assay using Roche Modular. KIF6 and ApoE genotyping were processed using real-time PCR (Berkeley Heart Lab, Berkeley, CA).

### 2.3. Statistical Analysis

Chi-square analyses were used to assess differences in frequency for gender, disease status (AD versus aMCI), 719Arg carrier status, and ApoE *ε*4 carrier status. For the Chi-square analyses, a Bonferroni adjusted *P*-value of 0.025 was used to correct multiple comparisons. Log transformations were performed on the raw data for tHcy and CRP in order to normalize their distributions. For log-transformed tHcy and CRP values, geometric means with 95% confidence intervals are reported. Group differences between 719Arg carriers and noncarriers for tHcy and CRP were assessed using a two-sample *t*-test. Additional two-sample *t*-tests were carried out for comparisons of gender, ApoE *ε*4 carrier status, and cholesterol drug (statin) use status on tHcy and CRP.

Logistic regression was used to analyze the associations between tHcy and CRP with 719Arg carrier status in order to provide an estimate of effect size and to provide a more practical interpretation of the associations than the two-sample *t*-tests can provide. For these analyses, the sample was dichotomized into elevated and normal groups using recommended clinical guidelines [35] for tHcy and CRP. Individuals with tHcy levels that were ≥14 *μ*mol/L were classified as elevated while individuals with CRP levels that were >3.0 mg/L were classified as elevated. For the logistic models, elevated/normal status for tHcy and CRP was used as the outcome variable with 719Arg carrier status as the predictor variable. Although dichotomizing continuous variables can result in a loss of statistical power, when the cut-point used has clinical significance this approach is justified [36]. Age, gender, ApoE *ε*4 carrier status, and statin use were used as covariates.

Four different logistic regression models were carried out for both tHcy and CRP using elevated versus normal status as the outcome (8 models total). The first model used only 719Arg carrier status as the predictor variable in order to assess the crude associations with tHcy and CRP. The second model included ApoE *ε*4 carrier status, age, and gender in order to account for their effects. The third model adjusted for statin use in addition to age, gender, and ApoE *ε*4 carrier status. The fourth model used a multiplicative interaction term for 719Arg carrier status and ApoE *ε*4 carrier status (719Arg × ApoE *ε*4) as the predictor variable while adjusting for age, gender, and statin use. The fourth model was intended to be an exploratory analysis to further rule out the effect of ApoE *ε*4 carrier status on the association between 719Arg carrier status with tHcy and CRP.

## 3. Results

The study sample was comprised of 82 females and 93 males with an average age of 77.92 ± 8.23 years. Clinical and demographic characteristics of the study sample are displayed in Table 1. For tHcy and CRP, the means and standard deviations of the raw values are reported in addition to the geometric means and their 95% confidence intervals. There was no significant association between 719Arg carrier status, ApoE *ε*4 carrier status, gender, or statin use. There was no significant difference for age or CRP between 719Arg carriers and noncarriers; however, 719Arg carriers had significantly lower tHcy levels. We also analyzed tHcy and CRP by gender, ApoE *ε*4 carrier status, and statin use status (Table 2). The only significant differences noted were for gender and tHcy where males were significantly higher and for ApoE *ε*4 carrier status and CRP where *ε*4 noncarriers were significantly higher than carriers.

A post-hoc power analysis for the two-sample *t*-tests found that our sample was large enough to detect a medium effect size (*d* = 0.50) with 90% statistical power [37]. An additional power analysis for the logistic regression analyses demonstrated that our sample achieved 91% power.

Using methods described by Rodriguez et al. [38], we determined whether the frequency of KIF6 and ApoE genotypes were consistent with the Hardy-Weinberg equilibrium. The frequency of KIF6 genotypes did not violate the Hardy-Weinberg equilibrium (*χ* ^2^ = 1.44, df = 2, *P* = 0.49). The genotype frequency for ApoE *ε*4 noncarriers did not violate the Hardy-Weinberg equilibrium (*χ* ^2^ = 0.30, df = 2, *P* = 0.86). However, the genotype frequency for ApoE *ε*4 carriers did violate the Hardy-Weinberg Equilibrium (*χ* ^2^ = 40.09, df = 2, *P* < 0.001). The latter is likely due to the fact AD studies tend to have a greater proportion of ApoE *ε*4 carriers when compared to the genotype's population prevalence [39]. Specifically, the frequency of individuals with the 3/4 genotype (*n* = 80) was substantially greater than the frequencies of the 2/4 (*n* = 4) and 4/4 (*n* = 15) genotypes. The prevalence of the 2/4 genotype (2%) in our study is consistent with population prevalence estimates proposed by Raber et al. [40]; however, the prevalence of the 3/4 (46%) and 4/4 (9%) genotypes in our study are higher than what would be expected in the general population. Given the *ε*4 genotype's well known association with AD, the violation of the Hardy-Weinberg Equilibrium described above is not surprising. Fisher exact test results found that there was no significant difference in genotype frequency between aMCI and AD patients for KIF6 (*P* = 0.09) and ApoE (*P* = 0.99).

Results of the logistic regression models for tHcy and CRP are displayed in Table 3. 719Arg carrier status showed no significant association with elevated CRP but did demonstrate a significant association with elevated tHcy status even after adjusting for ApoE *ε*4 carrier status, age, gender, and statin use. The association between 719Arg carrier status and elevated tHcy showed that 719Arg carriers were less likely to have elevated tHcy than noncarriers. The models that tested the KIF6 × ApoE *ε*4 interaction showed no significant association with elevated tHcy or elevated CRP. We carried out additional logistic regression analyses to assess the associations for ApoE *ε*4 carrier status with tHcy and CRP as the outcomes. Both analyses yielded nonsignificant results: tHcy [OR = 0.99 (0.54, 1.80), *P* = 0.97]; CRP [OR = 0.58 (0.30, 1.16), *P* = 0.12].

## 4. Discussion

This study is the first to assess the associations between KIF6 719Arg carrier status with tHcy and CRP in a sample of aMCI and AD patients. The results of this study showed that 719Arg carriers had significantly lower tHcy levels relative to noncarriers. Also, 719Arg carriers were also less likely to have elevated tHcy levels relative to noncarriers. Even after adjusting for age, gender, ApoE *ε*4 carrier status, and statin use, the magnitude of the association did not change significantly. The nonsignificant interaction between KIF6 and ApoE on tHcy suggests that the association between KIF6 and tHcy is independent of ApoE. The importance of this finding is highlighted by previous studies demonstrating associations between ApoE and tHcy [28, 41] and suggests that KIF6 might be involved in AD-related cardiovascular pathways that are independent of those associated with ApoE.

Although 719Arg carrier status and CRP were not significantly associated, the direction of the association in this study suggests that 719Arg carriers are more likely to have elevated CRP levels. Even though this association was not statistically significant, it is still interesting to note as ApoE *ε*4 carrier status and statin use were accounted for since both have been associated with decreased CRP [14, 28, 42, 43].

The mechanism by which KIF6 may affect tHcy is unclear as previous studies of KIF6 719Arg carrier status have focused primarily on cholesterol levels, statin use, and incidence of cardiovascular events [1–6]. Based on the results of this study, it is unlikely that statin use has any effect on tHcy levels since there was no significant difference between statin users and nonusers. Further, all of our primary analyses controlled for statin use so it reasonable to conclude that our findings for tHcy and 719Arg carrier status are independent of statin use. However, Maitland-van der Zee et al. [44] found that the presence of certain MTHFR polymorphisms can enhance the beneficial effect of pravastatin in terms of cardiovascular risk reduction and suggest that this association might be due to a statin-induced lowering of tHcy. In a review by Dierkes et al. [45], it was concluded that statin use is not associated with tHcy reduction based on the results of several prospective studies.

An area of interest for future studies would be to assess the role of the MTHFR gene in the relationship between tHcy and KIF6. Since MTHFR is responsible for the metabolism of tHcy, it would be interesting to see if MTHFR polymorphisms interact with KIF6 in terms tHcy levels. Recent evidence has suggested that the C677T polymorphism of MTHFR is associated with AD independently of ApoE *ε*4 carrier status [46] while Anello et al. [47] reported that the effect of homocysteine as a risk factor for AD was exacerbated when both ApoE *ε*4 and the MTHFR 677T allele were present. Given the results of our study, it would be interesting to see if positive carrier status for KIF6 719Arg mitigates this association since 719Arg carriers in our study had lower overall tHcy levels and a lower risk of having elevated tHcy relative to 719Arg noncarriers. This finding may also be of value to studies in the area of cardiology given the association between elevated homocysteine and cardiovascular disease [48].

Since increased tHcy has been linked to the pathophysiology of AD [49], the role of KIF6 in this pathway may be of importance. Although it is unlikely that KIF6 plays a direct role in AD pathogenesis, it is possible that KIF6 is responsible for certain mediating effects along tHcy-related pathways for AD. The results of this study suggest that carriers of the KIF6 719Arg allele have a preferential tHcy profile that may provide some level of protection along AD-related cardiovascular pathways involving tHcy.

## Figures and Tables

**Table 1:** Demographic and clinical characteristics.

	719Arg carriers	719Arg noncarriers	*P* value
*N*	106	69	—
Males/females (*n*)	54/52	39/30	0.47
ApoE *ε*4 carrier/noncarrier (*n*)	59/47	40/29	0.76
Statin use/no statin use (*n*)	59/47	46/23	0.15
Age	77.18 ± 8.58	79.06 ± 7.58	0.13
tHcy (*μ*mol/L)^†^	13.64 ± 4.59	15.12 ± 4.42	—
CRP (mg/L)^†^	4.32 ± 8.49	3.46 ± 8.60	—
tHcy (*μ*mol/L)^‡^	12.94 (12.16, 13.77)*	14.49 (13.46, 15.56)*	0.02
CRP (mg/L)^‡^	1.47 (1.11, 1.96)*	1.21 (0.90, 1.63)*	0.37

Mean ± standard deviation.

^†^Raw scores.

^‡^Log transformed scores.

*Geometric mean (95% confidence interval).

**Table 2:** Analysis of confounding factors for tHcy and CRP.

	tHcy (*μ*mol/L)	CRP (mg/L)
ApoE *ε*4 carrier status	Carrier: 13.43 (12.68, 14.22)	Carrier: 1.08 (0.80, 1.44)
	Noncarrier: 13.68 (12.62, 14.83)	Noncarrier: 1.86 (1.40, 2.47)
	*P* = 0.71	*P* = 0.01

Gender	Males: 14.32 (13.52, 15.21)	Males: 1.15 (0.84, 1.56)
	Females: 12.68 (11.78, 13.68)	Females: 1.66 (1.25, 2.20)
	*P* = 0.01	*P* = 0.08

Statin use status	Statin—yes: 13.15 (12.05, 14.32)	Statin—yes: 1.57 (1.09, 2.28)
	Statin—no: 13.80 (13.06, 14.59)	Statin—no: 1.24 (0.97, 1.59)
	*P* = 0.32	*P* = 0.28

Geometric mean (95% confidence interval).

*P*: *P* value.

**Table 3:** KIF6 719Arg carrier status association with elevated tHcy and CRP.

	tHcy	CRP
Model 1—no adjustment	0.41 (0.22, 0.75) *P* = 0.004	1.86 (0.90, 3.87) *P* = 0.10

Model 2—adjusted for ApoE *ε*4, age, and gender	0.43 (0.23, 0.82) *P* = 0.01	1.90 (0.90, 4.01) *P* = 0.10

Model 3—adjusted for ApoE *ε*4, age, gender, and statin use	0.44 (0.23, 0.83) *P* = 0.01	1.80 (0.85, 3.83) *P* = 0.13

Model 4—KIF6 × ApoE *ε*4 interaction adjusted for age, gender, and statin use	0.59 (0.30, 1.14) *P* = 0.12	0.80 (0.38, 1.68) *P* = 0.55

Odds ratio (95% confidence interval).

*P* value.

## References

[1] Iakoubova OA, Tong CH, Rowland CM (2008). Association of the Trp719Arg polymorphism in kinesin-like protein 6 with myocardial infarction and coronary heart isease in 2 prospective trials. The CARE and WOSCOPS Trials. *Journal of the American College of Cardiology*.

[2] Iakoubova OA, Robertson M, Tong CH (2010). KIF6 Trp719Arg polymorphism and the effect of statin therapy in elderly patients: results from the PROSPER study. *European Journal of Cardiovascular Prevention and Rehabilitation*.

[3] Li Y, Iakoubova OA, Shiffman D, Devlin JJ, Forrester JS, Superko HR (2010). KIF6 polymorphism as a predictor of risk of coronary events and of clinical event reduction by statin therapy. *American Journal of Cardiology*.

[4] Iakoubova OA, Sabatine MS, Rowland CM (2008). Polymorphism in KIF6 Gene and Benefit From Statins After Acute Coronary Syndromes. Results From the PROVE IT-TIMI 22 Study. *Journal of the American College of Cardiology*.

[5] Shiffman D, Chasman DI, Zee RYL (2008). A kinesin family member 6 variant is associated with coronary heart disease in the women’s health study. *Journal of the American College of Cardiology*.

[6] Shiffman D, O’Meara ES, Bare LA (2008). Association of gene variants with incident myocardial infarction in the cardiovascular health study. *Arteriosclerosis, Thrombosis, and Vascular Biology*.

[7] Hopewell JC, Parish S, Clarke R (2011). No impact of KIF6 Genotype on vascular risk and statin response among 18,348 randomized patients in the heart protection study. *Journal of the American College of Cardiology*.

[8] Assimes TL, Hólm H, Kathiresan S (2011). Lack of association between the trp719Arg polymorphism in kinesin-like protein-6 and coronary artery disease in 19 case-control studies. *Journal of the American College of Cardiology*.

[9] Sabbagh M, Malek-Ahmadi M, Levenson I, Sparks DL (2013). KIF6 719Arg allele is associated with statin effects on cholesterol levels in amnestic mild cognitive impairment and Alzheimer's disease patients. *Journal of Alzheimers Disease*.

[10] Jick H, Zornberg GL, Jick SS, Seshadri S, Drachman DA (2000). Statins and the risk of dementia. *Lancet*.

[11] Cramer C, Haan MN, Galea S, Langa KM, Kalbfleisch JD (2008). Use of statins and incidence of dementia and cognitive impairment without dementia in a cohort study. *Neurology*.

[12] Panza F, Solfrizzi V, D’Introno A (2009). Higher total cholesterol, cognitive decline, and dementia. *Neurobiology of Aging*.

[13] Solfrizzi V, D’Introno A, Colacicco AM (2006). Circulating biomarkers of cognitive decline and dementia. *Clinica Chimica Acta*.

[14] Haan MN, Miller JW, Aiello AE (2007). Homocysteine, B vitamins, and the incidence of dementia and cognitive impairment: results from the Sacramento Area Latino Study on Aging. *American Journal of Clinical Nutrition*.

[15] Seshadri S, Beiser A, Selhub J (2002). Plasma homocysteine as a risk factor for dementia and Alzheimer’s disease. *New England Journal of Medicine*.

[16] Kravitz BA, Corrada MM, Kawas CH (2009). Elevated C-reactive protein levels are associated with prevalent dementia in the oldest-old. *Alzheimer’s and Dementia*.

[17] Engelhart MJ, Geerlings MI, Meijer J (2004). Inflammatory proteins in plasma and the risk of dementia: the rotterdam study. *Archives of Neurology*.

[18] Schmidt R, Schmidt H, Curb JD, Masaki K, White LR, Launer LJ (2002). Early inflammation and dementia: a 25-year follow-up of the Honolulu-Asia Aging Study. *Annals of Neurology*.

[19] Troen A, Rosenberg I (2005). Homocysteine and cognitive function. *Seminars in Vascular Medicine*.

[20] Hasegawa T, Ukai W, Jo DG (2005). Homocysteic acid induces intraneuronal accumulation of neurotoxic A*β*42: implications for the pathogenesis of Alzheimer’s disease. *Journal of Neuroscience Research*.

[21] Irizarry MC, Gurol ME, Raju S (2005). Association of homocysteine with plasma amyloid *β* protein in aging and neurodegenerative disease. *Neurology*.

[22] Eikelenboom P, Hoozemans JJ, Veerhuis R, van Exel E, Rozemuller AJ, van Gool WA (2012). Whether, when and how chronic inflammation increases the risk of developing late-onset Alzheimer's disease. *Alzheimers Research and Therapy*.

[23] Bi BT, Lin HB, Cheng YF (2012). Promotion of *β*-amyloid production by C-reactive protein and its implications in the early pathogenesis of Alzheimer’s disease. *Neurochemistry International*.

[24] Kok EH, Alanne-Kinnunen M, Isotalo K (2011). CRP gene variation affects early development of Alzheimer’s disease-related plaques. *Journal of Neuroinflammation*.

[25] O'Bryant SE, Waring SC, Hobson V (2010). Decreased C-reactive protein levels in Alzheimer disease. *Journal of Geriatric Psychiatry and Neurology*.

[26] Roberts RO, Geda YE, Knopman DS (2009). Association of C-reactive protein with mild cognitive impairment. *Alzheimer’s and Dementia*.

[27] Haan MN, Aiello AE, West NA, Jagust WJ (2008). C-reactive protein and rate of dementia in carriers and non carriers of Apolipoprotein APOE4 genotype. *Neurobiology of Aging*.

[28] Ravaglia G, Forti P, Maioli F (2006). Apolipoprotein E e4 allele affects risk of hyperhomocysteinemia in the elderly. *American Journal of Clinical Nutrition*.

[29] Ridker PM (2005). C-reactive protein, inflammation, and cardiovascular disease: clinical update. *Texas Heart Institute Journal*.

[30] Wald DS, Law M, Morris JK (2002). Homocysteine and cardiovascular disease: evidence on causality from a meta-analysis. *British Medical Journal*.

[31] Grammas P (2011). Neurovascular dysfunction, inflammation and endothelial activation: implications for the pathogenesis of Alzheimer’s disease. *Journal of Neuroinflammation*.

[32] Hofman A, Ott A, Breteler MMB (1997). Atherosclerosis, apolipoprotein E, and prevalence of dementia and Alzheimer’s disease in the Rotterdam Study. *Lancet*.

[33] McKhann G, Drachman D, Folstein M (1984). Clinical diagnosis of Alzheimer’s disease: report of the NINCDS-ADRDA work group under the auspices of Department of Health and Human Services Task Force on Alzheimer’s disease. *Neurology*.

[34] Petersen RC, Smith GE, Waring SC, Ivnik RJ, Tangalos EG, Kokmen E (1999). Mild cognitive impairment: clinical characterization and outcome. *Archives of Neurology*.

[35] Clinical Implications Reference Manual. http://www.bhlinc.com/clinicians/clinical-references/reference-manual/.

[36] Ragland DR (1992). Dichotomizing continuous outcome variables: dependence of the magnitude of association and statistical power on the cutpoint. *Epidemiology*.

[37] Faul F, Erdfelder E, Lang AG, Buchner A (2007). G∗power 3: a flexible statistical power analysis program for the social, behavioral, and biomedical sciences. *Behavior Research Methods*.

[38] Rodriguez S, Gaunt TR, Day INM (2009). Hardy-Weinberg equilibrium testing of biological ascertainment for Mendelian randomization studies. *American Journal of Epidemiology*.

[39] Ward A, Crean S, Mercaldi CJ (2012). Prevalence of Apolipoprotein E4 genotype and homozygotes (APOE e4/4) among patients diagnosed with alzheimer’s disease: a systematic review and meta-analysis. *Neuroepidemiology*.

[40] Raber J, Huang Y, Ashford JW (2004). ApoE genotype accounts for the vast majority of AD risk and AD pathology. *Neurobiology of Aging*.

[41] Minagawa H, Watanabe A, Akatsu H (2010). Homocysteine, another risk factor for Alzheimer disease, impairs apolipoprotein E3 function. *Journal of Biological Chemistry*.

[42] Ridker PM, Rifai N, Pfeifer MA, Sacks F, Braunwald E (1999). Long-term effects of pravastatin on plasma concentration of C-reactive protein. *Circulation*.

[43] Ridker PM, Cannon CP, Morrow D (2005). C-reactive protein levels and outcomes after statin therapy. *New England Journal of Medicine*.

[44] Maitland-van der Zee AH, Lynch A, Boerwinkle E (2008). Interactions between the single nucleotide polymorphisms in the homocysteine pathway (MTHFR 677C>T, MTHFR 1298 A>C, and CBSins) and the efficacy of HMG-CoA reductase inhibitors in preventing cardiovascular disease in high-risk patients of hypertension: the GenHAT study. *Pharmacogenetics and Genomics*.

[45] Dierkes J, Luley C, Westphal S (2007). Effect of lipid-lowering and anti-hypertensive drugs on plasma homocysteine levels. *Vascular Health and Risk Management*.

[46] Wang B, Jin F, Kan R (2005). Association of MTHFR gene polymorphism C677T with susceptibility to late-onset Alzheimer’s disease. *Journal of Molecular Neuroscience*.

[47] Anello G, Guéant-Rodríguez RM, Bosco P (2004). Homocysteine and methylenetetrahydrofolate reductase polymorphism in Alzheimer's disease. *Neuroreport*.

[48] Refsum H, Ueland PM, Nygård O, Vollset SE (1998). Homocysteine and cardiovascular disease. *Annual Review of Medicine*.

[49] Seshadri S (2006). Elevated plasma homocysteine levels: risk factor or risk marker for the development of dementia and Alzheimer’s disease?. *Journal of Alzheimer’s Disease*.

